# D-Cycloserine as an augmentation strategy for cognitive behavioral therapy of anxiety disorders

**DOI:** 10.1186/2045-5380-3-11

**Published:** 2013-06-15

**Authors:** Stefan G Hofmann, Jade Q Wu, Hannah Boettcher

**Affiliations:** 1 Department of Psychology, Boston University, Boston, MA, 02215-2002, USA

**Keywords:** Anxiety disorder, Cognitive behavioral therapy, Glutamate, D-cycloserine, NMDA, Psychotherapy, Pharmacotherapy

## Abstract

The goal of this review is to examine the clinical studies on d-cycloserine, a partial glutamatergic N-methyl-D-aspartate agonist, as an augmentation strategy for exposure procedures during cognitive behavioral therapy for anxiety disorders. Although cognitive behavioral therapy and anxiolytic medications are more effective than placebo for treating anxiety disorders, there is still considerable room for further improvement. Traditional combination strategies typically yield disappointing results. However, recent studies based on translational research have shown promise to augment the neural circuitry underlying fear extinction with pharmacological means. We discuss the current state of the literature, including inconsistencies of findings and issues concerning the drug mechanism, dosing, and dose timing. D-cycloserine is a promising combination strategy for cognitive behavioral therapy of anxiety disorders by augmenting extinction learning. However, there is also evidence to suggest that d-cycloserine can facilitate reconsolidation of fear memory when exposure procedures are unsuccessful.

## Review

### Background

Anxiety disorders are among the most common mental health problems [1] and are associated with significantly reduced quality of life [2]. The most effective psychological treatment for anxiety disorders is cognitive behavioral therapy (CBT). A meta-analysis of placebo-controlled trials of CBT yielded an average effect size of 0.73 [3,4], suggesting that many patients do not improve after an adequate course of CBT. Similarly, traditional pharmacological treatments for anxiety disorders, which include monoamine oxidase inhibitors, selective serotonin reuptake inhibitors, tricyclic antidepressants, and benzodiazepines, are only modestly effective [5-7] and the understanding of the precise psychobiological mechanism of these treatments remains limited [7]. In some cases, combining anti-anxiety medication with psychotherapy can even cause adverse effects in the long term, such as in the case of benzodiazepines [8].

Given the lackluster success of CBT and anxiolytic medications, it seems reasonable to combine both treatment modalities in order to maximize treatment efficacy. For example, it may be possible that anxiolytic pharmacotherapy increases the ability for CBT principles to be learned. However, such combination strategies typically yield disappointing results as shown in a meta-analytic review that examined all randomized placebo-controlled clinical trials comparing combination treatment (i.e., CBT plus pharmacotherapy) with CBT plus placebo across the anxiety disorders [9]. Although there are some studies that seem to suggest that medications can support CBT, the general results revealed only modest benefits of combined interventions immediately following treatment and for only some anxiety disorders. No benefit was observed for CBT plus pharmacology over CBT plus pill placebo at the 6-month follow-up. For example, a well-designed clinical trial examined the relative efficacy of fluoxetine, CBT, pill placebo, CBT combined with fluoxetine, and CBT combined with pill placebo for social anxiety disorder [10]. The response rates in the intention-to-treat sample were 50.9% (fluoxetine), 51.7% (CBT), 54.2% (CBT/fluoxetine), 50.8% (CBT/placebo), and 31.7% (placebo). Very similar efficacy rates are reported for other classes of drugs and other forms of anxiety disorders.

The reasons for these disappointing findings are not well understood. It is possible that the affect-modulating properties of anxiolytic drugs may interfere with CBT by providing conditions for state-dependent learning, thereby creating a unique internal context due to their efficacy at modulating affect or side effects, which make the presence of these medications discriminable to patients [11]. It is also possible that patients attribute the fear reduction during CBT to the pill rather than the exposure practices during a combination therapy, which may negatively affect their perception of self-efficacy with regard to the treatment gains [12]. Finally, CBT and anxiolytic drugs may operate via different and non-additive mechanisms that are slow and suboptimal.

In recent years, a new approach of combination treatment has emerged. This approach evolved from translational research on the neurocircuitry of extinction learning. Instead of combining CBT with pharmacological agents acting as anxiolytics, CBT is combined with pharmacological agents acting as cognitive enhancers to augment certain learning processes (e.g., cortisol, propranolol, yohimbine) [13]. Some studies have identified d-cycloserine (DCS) as a particularly promising pharmacological candidate for augmenting CBT.

Since the publication of previous reviews of evolving research on DCS as an enhancer to exposure-based therapies [12,14], a number of important new preclinical studies and clinical trials have been added to the literature. These findings may shed light on the mechanism of the observed effects. Although some of these recent studies have failed to replicate previous successful trials, a careful comparison between the various trials provides some valuable insight into how and when DCS should be used. In this review, we will first briefly review the theoretical basis for DCS as a pharmacological enhancer, then summarize the clinical trials testing its efficacy in various anxiety disorders (i.e., specific phobia, social anxiety disorder, obsessive compulsive disorder, panic disorder, and post-traumatic stress disorder) and finally discuss the clinical implications of these findings.

## Theoretical basis

### Learning processes

Exposure therapy is based on research on fear conditioning and extinction learning [15-17]. For example, Mowrer [17] and others (e.g., Rescorla [18], Rachman [19]) proposed that fears are acquired through repeated presentations of a neutral stimulus (conditioned stimulus; CS) and a pain-producing or fear-eliciting stimulus (unconditioned stimulus; US). The strength of the fear response is determined by the association between the CS and US, and the intensity of the unconditioned response. The repeated presentation of the CS in the absence of the US leads to extinction, the gradual decrease of the conditioned response. This process has been regarded as an essential component of exposure therapy in humans from the early beginnings of experimental studies in psychology [20] to the contemporary field of neuroscience [21,22].

Extinction is not simply an unlearning or forgetting but rather a new form of learning that changes the CS-US contingency in such a way that the CS no longer signals an aversive event and thereby inhibits the expression of the fear response [23-26]. Evidence for this notion comes from experiments showing spontaneous recovery of a previously conditioned fear response [27], renewal [28], and reinstatement of fear [29-32].

### Cognitive processes

Contemporary learning theory suggests that conditioning and extinction result from combinations of cognitive representations about meaningful cues (the US and CS) and the contexts in which they associate [23-25,33]. When two stimuli are presented together, an association forms between them. Thereafter, representation of the CS activates representation of the US, leading to the conditioned response. During extinction, the association between the CS and US is weakened with each presentation of the CS that fails to be accompanied by the US. As a result, the CS no longer predicts an aversive event. In other words, extinction learning and exposure therapy is associated with changes in US-CS expectancy, which involves cognitive processes [34]. Further, other cognitive changes contribute to effective extinction; for example, it has been shown that informing subjects that the US will not accompany the CS leads to decreased responsivity to the CS [35].

### Biological processes

Fear extinction is associated with activation in the medial prefrontal cortex in rats [36] and humans [37] and also the basolateral nucleus of the amygdala [38,39]. On the neuronal level, extinction learning requires long-term potentiation (LTP), which is the process by which the repeated firing of synchronous neurons strengthens the connection between them over time.

An important neurotransmitter involved in extinction learning is glutamate, which is an excitatory neurotransmitter in the mammalian brain. An important receptor complex involved in this process is the N-methyl-D-aspartate (NMDA) receptor. Activation of the NMDA receptor requires binding of both glutamate and the co-agonist glycine. This activation can then lead to LTP, which is the neuronal basis for learning, including fear conditioning [40] and extinction learning [36-39].

It has been shown that D-4-amino-3-isoxazolidone (d-cycloserine, DCS), which is a partial agonist at the glycine recognition site of the glutamatergic NMDA receptor, can augment extinction learning in animals [41,42]. Walker and colleagues conducted the first study examining the effects of DCS on extinction learning in rats. In a paradigm assessing extinction by measuring fear-potentiated startle to a conditioned stimulus, the authors found that extinction was more successful when preceded by administration of DCS. Other studies replicated this finding [42,43].

In contrast to the animal literature, the DCS-augmentation effect for extinction learning and exposure therapy in humans is less consistent. As we will discuss in more detail below, some of the inconsistent findings might be due to the fact that DCS not only augments extinction learning but also enhances reconsolidation of fear memory in animals. More specifically, animal studies have shown that NMDA antagonists impair the reconsolidation of fear memories [44-46] whereas DCS enhances reconsolidation of fear memory in humans when administered into the basolateral amygdala [47]. Therefore, if fear does not sufficiently decrease during exposure therapy, fear memory reconsolidation may occur and DCS can facilitate this counter-therapeutic process. In other words, DCS can make “good” exposures better and “bad” exposures worse. It should be noted, however, that this is a post-hoc, yet plausible, explanation for some of the negative findings reported in some clinical trials.

It is further important to note that in animal studies, DCS only revealed positive effects on learning when administered in isolated (i.e., acute) dosing [48]. When administered chronically (i.e., repeatedly over an extended period of time), the NMDA receptor complex can become desensitized, leading DCS to effectively work as an NMDA antagonist [49]. Similarly, long-term exposure to all major classes of antidepressants (including selective serotonin reuptake inhibitors, tricyclics, and monoamine reuptake inhibitors) are associated with neurochemical changes at the glycine binding site of the NMDA receptor complex, altering the action of DCS [50,51]. Similar effects have been found after electroconvulsive therapy [52].

Therefore, DCS can augment extinction learning in animals and exposure procedures in humans, but it can also enhance fear reconsolidation. This can occur if exposure procedures did not result in a sufficient reduction of fear. Moreover, there is good evidence to suggest that chronic administration of DCS, as well as concurrent or past treatments with anxiolytic medication and electroconvulsive therapy can significantly impair or even counter - at least temporarily - the augmentation effects of DCS for extinction learning in animals and exposure therapy in humans. These issues need to be considered when reviewing the clinical trial literature of DCS augmentation for anxiety disorders. A summary of this literature is presented in Table 1.

**Table 1:** Characteristics and results of clinical trials of DCS-augmented CBT for anxiety disorders

**Study**	**Diagnosis**	**N**	**DCS Dose (mg)**	**Dosing timing (hrs before exposure)**	**# of DCS doses**	**CBT type (# of exposure sessions)**	**Primary measures**	**Main results (DCS vs. placebo)**
Ressler et al., 2004 [53]	Acrophobia	27	50 or 500	2-4	2	VRE (2)	SUDS, skin conductance, clinical self-reports	Greater subjective improvement (*p < .001)*; greater decrease in skin conductance fluctuations (*p < .05);* greater clinical improvement (*p < .02)* effects were maintained at 3-month follow-up
Guastella et al., 2007 [54]	Sub-threshold spider phobia	63	50	2-3	1	Exposure (1)	SUDS, heart rate	No DCS effects
Tart et al., 2013 [55]	Acrophobia	29	50	−0.5^a^	2	VRE (2)	SUDS, CGI-I	No overall DCS effects.
Smits et al., 2013 [56]								DCS effect on CGI-I significantly moderated by fear level at end of exposure session (*b = −.05, p < .01*); low levels of fear predicted greater symptom improvement
Nave et al., 2012 [57]	Snake phobia	20	50	1	1	Exposure (1)	Snake Questionnaire	No overall DCS effects.
								DCS group reached top of fear hierarchy more quickly (p < .05)
Hofmann et al., 2006 [58]	SAD	27	50	1	5	Individual/group CBT, emphasis on exposure (5)	SPAI, LSAS	Greater symptom improvement (p’s < .02); effects maintained at 1-month follow-up
Guastella et al., 2008 [59]	SAD	56	50	1	4	Group CBT, emphasis on exposure (4)	SPAI, LSAS	Greater symptom improvement over time (*p = .*002).
Rodebaugh et al., in press [60]	SAD	34	250	0	1	Exposure (2)	SUDS	Greater reduction in subjective distress between two exposure sessions (*d* = 1.06)
Hofmann et al., in press [61]	SAD	169	50	1	5	CBT (5)	LSAS, SPDS	Faster symptom improvement, global illness severity and remission status (*p’s* < .05)
Wilhelm et al., 2008 [62]	OCD	23	100	1	10	Exposure-based behavior therapy (10)	YBOCS	No overall DCS group effect; significant time by group interaction (*p* = .02), with greater symptom improvement in DCS group at mid-treatment.
Chasson et al. 2010 [63]								Re-analysis showed DCS increased speed of symptom improvement sixfold during the first half of treatment.
Kushner et al., 2007 [64]	OCD	25	125	2	10	ERP (10)	YBOCS	Lower drop-out rate (*p <* .05); symptoms improved more quickly during first 4 sessions (*p =* .02*, d =* .77)
Storch et al., 2007 [65]	OCD	24	250	4	12	ERP (12)	YBOCS	No DCS effects.
Storch et al., 2010 [66]	Pediatric OCD (ages 8–17)	30	25 or 50	1	7	ERP (7)	CYBOCS, CGI-S, ADIS-CSR	Small-to-moderate DCS effects (*d* = .31-.47)
Otto et al., 2010 [67]	PD, PDA	31	50	1	3	Brief CBT (3)	PDSS, CGI-S	Greater symptom and severity reduction (*p = .*01*, d =* 1.11), maintained at follow-up (*p* < .05)
Siegmund et al., 2011 [68]	PDA	39	50	1	3	CBT (3)	PAS, CGI	No overall DCS effects; statistical trend (p = 0.075) in severely ill patients that DCS accelerated symptom reduction
de Kleine et al., 2012 [69]	PTSD	67	50	1	7-9	Prolonged Exposure (7–9)	CAPS	No overall DCS effects; DCS group more likely to show response (odds ratio 2.83, 95 % confidence interval [CI] 1.05–7.61).
Litz et al., 2012 [70]	PTSD	26	50	0.5	4	Brief CBT (4)	CAPS, PTSD Checklist	DCS associated with poorer outcome on all measures

*ADIS-CSR* = Anxiety Disorders Interview Schedule-Clinical Severity Rating; *CAPS* = Clinician-Administered PTSD Scale; *CBT* = cognitive-behavioral therapy; *CGI* = Clinical Global Impression; *CGI-I* = Clinical Global Impression-Improvement; *CGI-S* = Clinicians’ Global Impressions of Severity; *CYBOCS* = Child Yale Brown Obsessive Compulsive Scale; *ERP* = exposure/ritual prevention *or* exposure and response prevention; *LSAS* = Liebowitz social anxiety scale; *OCD* = obsessive compulsive disorder; *PAS* = Panic and Agoraphobia Scale; *PD* = Panic disorder; *PDA* = Panic disorder with agoraphobia; *PDSS* = Panic Disorder Severity Scale; *PTSD* = posttraumatic stress disorder; *SAD* = social anxiety disorder; *SPDS* = social phobic disorder severity scale; *SPQ* = spider phobia questionnaire; *SPAI* = social phobia and anxiety inventory; *SUDS* = subjective units of discomfort; *VRE* = Virtual Reality Exposure; *YBOCS* = Yale Brown Obsessive Compulsive Scale.

^a^A DCS dosing timing of −0.5 hours indicates that DCS was administered 0.5 hours after the beginning of exposure.

## Clinical trials

We conducted a literature search for relevant clinical trials in PubMed and Google Scholar, and also through manual searches to identify relevant studies up until March 2013. A study was included if it was a human clinical trial that examined the efficacy of CBT for a DSM-IV anxiety disorder. We used the following three sets of search terms: (1) *DCS* or *d-cycloserine*; (2) *CBT* or *cognitive-behavioral* or variations thereof; and (3) *anxiety* or *anxious* or full names and abbreviations of DSM-IV anxiety disorders. The PubMed search identified 76 studies, of which 9 met the inclusion criteria. The Google Scholar search identified 7 additional trials and 2 articles reporting re-analysis of previous data. All trials included a placebo control group and employed a randomized design. The following discusses the studies of each of the target disorders.

### Specific phobia

The first clinical trial of DCS-augmented exposure therapy was conducted in patients with specific phobia [53]. This study employed a randomized, placebo-controlled double-blind design and recruited subjects with a DSM-IV diagnosis of acrophobia (i.e., fear of heights). All participants received two sessions of virtual reality exposure therapy. Those in the treatment group received either a 50 or 500 mg dose of DCS prior to exposure sessions, whereas individuals in the control group received a pill placebo. Results demonstrated significant symptom improvement in the DCS group at 1 week and 3 months following exposure therapy, with no difference between high and low dose groups. In addition to symptom reduction, participants in the DCS group also showed greater decreases in skin conductance fluctuations during exposures than those in the control group.

A study with spider fearful individuals [54] did not find such exposure-enhancing effects of DCS. However, the participants in this study did not meet the diagnostic threshold for spider phobia. Therefore, the exposure strategies alone might have effectively reduced fear in this subclinical population, leaving insufficient room for DCS enhancement. Therefore, it is possible that a ceiling effect obscured any potential treatment-enhancing effects of DCS.

A recent trial examined the DCS augmentation effect in treating a clinical sample of individuals with acrophobia [55]. Although this study used a very similar design as employed by Ressler and colleagues [53], the results showed that DCS did not augment exposure therapy in the total sample. However, a post-hoc reanalysis of this trial revealed that symptom reduction in the DCS group was dependent on fear experienced at the conclusion of the exposure session [56]. That is, when the session was successful (i.e., fear was low by the end), participants in the DCS group improved more than those in the control group. However, when the session was unsuccessful (i.e., fear was still high by the end), patients in the DCS group improved less than those in the control group.

Another recent study [57] recruited individuals with snake phobia and randomly assigned them to receive DCS or placebo prior to exposure sessions. Both groups responded to treatment, but the DCS group improved more quickly than the control group. Further, the DCS group had different ventromedial prefrontal activation in response to snake stimuli at 1-week follow-up, as compared to controls. The authors concluded that acute DCS administration, when used as an augmentation to exposure, resulted in lasting and qualitative changes in prefrontal activity.

### Social anxiety disorder

One of the first clinical trials examining the augmentation effect of DCS for CBT was conducted with patients with social anxiety disorder [58]. All patients underwent 5 sessions of exposure-based CBT and were randomized to receive either 50 mg DCS or a matching placebo one hour before each session. Exposures involved giving increasingly difficult speeches in front of a video camera, confederate or other group members. Results showed significantly greater symptom improvement in the DCS group as compared to the control group at post-treatment. Symptoms further improved at 1-month follow-up for the DCS group, and at a faster rate than for the control group, demonstrating extended treatment effects.

This study was replicated by an independent group of investigators using the identical design and treatment protocol with a larger sample of patients with social anxiety disorder [59]. Their weekly tracking data showed that, after the third exposure session, the DCS-augmented CBT group already showed significantly lower social anxiety symptoms than the placebo-augmented group.

An experimental study randomized patients with social anxiety disorder to receive 250 mg of DCS or placebo prior to a speech challenge test. All patients returned a second time 1 week later and were asked to give another speech. Remarkably, only a single pre-session administration of DCS already showed an augmentation effect in patients receiving DCS as compared to the placebo group [60].

Recently, the largest clinical trial of DCS augmentation of CBT for an anxiety disorder was completed. This trial, which recruited 169 patients with generalized social anxiety disorder, found that a 12-session course of CBT that included 5 DCS-augmented sessions did not result in better treatment response or remission rate relative to a placebo-augmented CBT group. However, the DCS-augmented CBT group showed a relatively greater rate of improvement [61].

### Obsessive-compulsive disorder

Four randomized, double-blind placebo-controlled trials of DCS augmentation have been conducted to examine the effects of DCS augmentation to exposure therapy for obsessive-compulsive disorder. Three of these trials were conducted with adults [62,64,65] and one with children [66]. All studies used exposure and response prevention (ERP; 4–12 sessions including introductory/psychoeducation sessions). Of the three adult trials, two demonstrated that DCS augmentation facilitated the efficiency of ERP therapy [62,64]. The trial that did not demonstrate an effect [65] administered 250 mg of DCS 4 hours prior to each of 12 CBT sessions. Notably, the design of this study is significantly different than the other DCS trials because the dosage, which was relatively high, was administered 4 hours instead of the more common 1 or 2 hours before CBT sessions. The pediatric obsessive-compulsive disorder trial [66] reported that the DCS-augmented CBT group showed small-to-moderate treatment effects over the CBT plus placebo group. However, these effects did not reach the level of statistical significance. This trial administered 25 or 50 mg of DCS 1 hour before each of 7 CBT sessions.

It is difficult to directly compare these trials on obsessive-compulsive disorder, because there were considerable differences in the dosing of DCS (ranging between 25 and 250 mg), dose timing (ranging between 1 hour and 4 hours before the exposures), administration schedule, study populations (children vs. adults) and even dropout rates (ranging between 6% and 35%; see Table 1).

The study by Wilhelm and colleagues [62] reported that although symptoms decreased for both groups, they were significantly lower in the DCS group than the control group at mid-treatment, but not at post-treatment. A re-analysis of these results [63] showed that treatment effects of exposure and response prevention were almost six times faster in the DCS group during the first half of treatment. This suggests that DCS augmentation did not necessarily amplify the treatment effects, but rather, made it more efficient by accelerating symptom reduction.

This pattern of symptom change is consistent with another trial that did not find group differences in symptom reduction overall [64]. This study reported a faster symptom improvement in the DCS group in sessions 4 through 6, whereas the placebo group improved faster in sessions 8 through 10. This may reflect the short-term efficiency-enhancing effects of DCS, which may be obscured by the disappointing results in the longer term.

### Panic disorder

A preliminary randomized, double-blind, placebo-controlled trial with panic disorder outpatients examined the augmentation effect of 50 mg doses of DCS, as compared to placebo, 1 hour before each of 5 weekly CBT sessions that emphasized interoceptive exposure [67]. Patients who received DCS-augmented exposures showed greater symptom reduction from baseline to post-treatment and to 1-month follow-up than patients in the control condition.

In contrast, a similar trial did not find an overall group effect of DCS augmentation [68]. However, the latter study reported an accelerated symptom reduction in severely ill patients, leading the authors to speculate that the lack of added benefit for patients in the DCS group was due to a floor effect for most patients. It should also be noted that this study employed 11 sessions of CBT, as opposed to the 5 used in the Otto et al. study [67]. This is an important difference, since chronic administration of DCS can counter and even reverse the augmentation effect.

### Post-traumatic stress disorder

So far, there are two published clinical trials examining the augmentation effect of DCS of CBT for post-traumatic stress disorder. The first clinical trial examining DCS as an augmentation for exposure therapy in patients with post-traumatic stress disorder failed to show any overall group differences [69]. However, the study reported some benefit for patients with more severe baseline symptoms. This pattern is consistent with previously described findings in panic disorder [68].

A second study reported a randomized, double-blind placebo-controlled trial of 50 mg DCS administered 30 minutes prior to imaginal exposure for 26 Afghanistan/Iraq veterans with combat-related post-traumatic stress disorder [70]. Those patients who received DCS reported *greater* symptoms at post-treatment as compared to patients in the placebo condition. It is unclear why these two DCS trials with post-traumatic stress disorder [69,70] reported conflicting findings. It may be possible that the inconsistency is due to methodological differences, including the gender of patients—the majority of patients were female in one study [69] and exclusively male in the other [70]. Moreover, the majority of patients in the former study were sexual trauma victims, while all patients in the latter study were combat-related trauma victims. Moreover, and more importantly, DCS has been shown not only to augment extinction learning, but also to enhance reconsolidation of fear memory. A close inspection of the distress ratings during the exposure trials in the Litz et al. study did, in fact, show evidence of less successful within-session extinction learning in the participants who received DCS [70]. Therefore, in the absence of sufficient extinction, DCS might have led to reconsolidation of the patients’ trauma memory, leading to worse outcomes than for patients who received placebo.

This notion is consistent with the earlier described study in acrophobia, the only trial where DCS was administered *after* exposure sessions [56]. This trial showed that DCS only exerted the augmentation effect over placebo when the exposure session was successful (i.e., fear was low by the end of the session). When the exposure session was not successful (i.e., the fear was still high at the end of the session), DCS patients improved *less* than those in the placebo group, consistent with the notion that DCS can augment both extinction learning and fear reconsolidation.

## Discussion

Basic research in animal extinction learning has recently been translated into novel treatment strategies for anxiety disorders. The result of this translational work has identified d-cycloserine as a possible cognitive enhancer to augment exposure strategies during CBT. This represents a fruitful example of translational research, whereby neuroscience has provided a novel, theoretically meaningful target for exploration in treatment realms. Following the initial excitement of early DCS augmentation treatment trials, a number of clinical trials have failed to replicate previous successes or have provided inconsistent results.

Specifically, DCS appears to augment a brief course of virtual reality exposure in patients with acrophobia [53], but did not show this augmentation effect in a replication study [54] or in subclinical spider-fearful individuals [54] and individuals with snake phobia [57]. Positive results were found in patients with social anxiety disorder [58-60]. The largest DCS augmentation trial to date showed that DCS was not associated with higher response or remission rates, as compared to placebo, but with a faster rate of improvement [61]. In two trials with adult obsessive-compulsive disorder patients, DCS augmented the efficacy of ERP [62,64]. One trial reported negative results, but this study was markedly different from the other trials in dosing and dose timing of DCS [65]. The only obsessive-compulsive disorder trial with a pediatric sample showed small to moderate, but non-significant, effects of DCS over placebo [66]. Mixed results were also found in two DCS trials of panic disorder. One of the trials reported that panic patients who received DCS prior to exposures showed greater symptoms reduction than those who received placebo prior to exposures [67]. A similar trial did not find an overall group effect, but the severely-ill patients showed accelerated symptom reductions [68]. Finally, two DCS trials were conducted with post-traumatic stress disorder. The first trial failed to show an overall group difference [69], but patients with more severe baseline symptoms benefitted more. In contrast, the second trial found that patients who received DCS reported greater symptoms at post-treatment than those who received placebo [70]. Several issues are important to consider when evaluating this literature.

First, it seems that DCS is more likely to accelerate than to amplify exposure procedures, because several clinical trials showed faster improvement in the DCS group in the short term, but equal improvements compared to placebo augmentation in the long term. Second, some trials found that DCS was only more effective than placebo in severely ill patients. This suggests that there might be important patient variables and other moderator variables that predict treatment outcome and response to DCS. Finally, dosing and dosing schedule are of critical importance. The first clinical trial administered single DCS doses of 50 mg or 500 mg, and reported no differences between these doses [33]. Since then, doses of 50, 100, and 125 mg have been used in subsequent studies and all doses appear adequate for enhancement of exposure therapy. However, the use of single 50 mg doses appears to minimize the risk of side effects and minimize concerns about tolerance to DCS, while showing relatively reliable effects when administered acutely. At higher doses (e.g., 500–1000 mg), especially when administered chronically, infrequent side effects occur (e.g., headache, drowsiness). Moreover, when administered more frequently or at higher doses, studies found DCS to have relatively weak results, sometimes even weaker than placebo.

In this context, timing is a particularly important clinical issue. As previously suggested [71], administering DCS too early may prevent important extinction learning processes from coinciding with the peak of the drug’s effect. In support of this idea, a meta-analysis of animal and human DCS studies showed that timing significantly predicted effect size, such that studies administering DCS immediately before or after exposure achieved greater effects than studies administering DCS multiple hours before exposure [14]. Of the clinical trials reviewed here, all but one study [56] administered DCS orally before the exposure sessions, usually 1 hour but sometimes 2 or more hours prior. This decision has been based on the fact that peak blood levels of DCS occur 4 to 6 hours after ingestion. However, it is possible that key extinction learning processes that may most benefit from DCS augmentation occur hours after the end of exposure sessions.

Moreover, timing is important because administering DCS prior to exposure introduces the possibility that DCS can augment reconsolidation of fear memories if the exposure session is unsuccessful. This observation is consistent with studies showing that DCS not only augments extinction learning but also enhances reconsolidation of fear memory in animals [47]. The study by Smits and colleagues showed that, despite the lack of overall DCS effects, post-session DCS administration did augment exposure sessions if the exposure sessions were successful [56]. Therefore, it could be argued that the decision to administer DCS should be made post-session, contingent on the level of fear reduced (i.e., extinction learning achieved) by the end of the session [56]. Another possibility is to include a clinical assay to detect patients who are likely to benefit from DCS-augmented treatment [60].

## Conclusion

A number of preclinical and clinical studies suggest that DCS may act as a cognitive enhancer during extinction learning and CBT. However, other studies report negative findings. Unanswered questions include the optimal dose and dose timing of DCS, possible interaction effects with other drugs, the long-term effects of DCS, and the specificity of the cognitive process that are targeted by the drug. Future studies examining the nuances of timing in DCS administration, as well as its interaction with the success of exposure sessions, can provide valuable new insights into the mechanisms of DCS and offer new means of treating some of the most common mental disorders. Once the mechanisms are clearly understood, future research will be able to explore other, and perhaps more effective, agents to augment specific processes during CBT and other interventions. Other important areas for future research include the post-session administration of DCS and the possible effect of DCS on reconsolidation of fear memory. If DCS, in fact, makes “good” exposures better and “bad” exposures worse, future investigators will need to identify individuals who are likely to benefit from DCS augmentation and those who are not, in line with a general move toward personalized medicine.

## Competing interests

The authors declare that they have no competing interest.

## Authors’ contributions

SGH wrote the initial and final draft of the manuscript; JQW carried out the literature review; QJW and HB assisted with writing. All authors read and approved the final manuscript.
